# Radiomics incorporating deep features for predicting Parkinson’s disease in ^123^I-Ioflupane SPECT

**DOI:** 10.1186/s40658-024-00651-1

**Published:** 2024-07-10

**Authors:** Han Jiang, Yu Du, Zhonglin Lu, Bingjie Wang, Yonghua Zhao, Ruibing Wang, Hong Zhang, Greta S. P. Mok

**Affiliations:** 1 Biomedical Imaging Laboratory (BIG), Department of Electrical and Computer Engineering, Faculty of Science and Technology, University of Macau, Avenida da Universidade, Macau, Macau SAR China; 2 PET-CT Center, Fujian Medical University Union Hospital, Fuzhou, China; 3 Center for Cognitive and Brain Sciences, Institute of Collaborative Innovation, University of Macau, Taipa, Macau SAR China; 4 Lauterbur Research Center for Biomedical Imaging, Shenzhen Institute of Advanced Technology, Chinese Academy of Sciences, Shenzhen, China; 5 State Key Laboratory of Quality Research in Chinese Medicine, Institute of Chinese Medical Sciences, University of Macau, Taipa, Macau SAR China; 6 Department of Nuclear Medicine and PET Center, The Second Affiliated Hospital of Zhejiang, University School of Medicine, 88 Jiefang Road, Zhejiang, 310009 Zhejiang China; 7 Institute of Nuclear Medicine and Molecular, Imaging of Zhejiang University, Hangzhou, China; 8 Key Laboratory of Medical Molecular Imaging of Zhejiang Province, Hangzhou, China; 9 College of Biomedical Engineering & Instrument Science, Zhejiang University, Hangzhou, China; 10 Key Laboratory for Biomedical Engineering of Ministry of Education, Zhejiang University, Hangzhou, China

**Keywords:** ^123^I-Ioflupane, SPECT, Parkinson’s disease, Radiomics, Deep learning, Deep feature

## Abstract

**Purpose:**

^123^I-Ioflupane SPECT is an effective tool for the diagnosis and progression assessment of Parkinson’s disease (PD). Radiomics and deep learning (DL) can be used to track and analyze the underlying image texture and features to predict the Hoehn-Yahr stages (HYS) of PD. In this study, we aim to predict HYS at year 0 and year 4 after the first diagnosis with combined imaging, radiomics and DL-based features using ^123^I-Ioflupane SPECT images at year 0.

**Methods:**

In this study, 161 subjects from the Parkinson’s Progressive Marker Initiative database underwent baseline 3T MRI and ^123^I-Ioflupane SPECT, with HYS assessment at years 0 and 4 after first diagnosis. Conventional imaging features (IF) and radiomic features (RaF) for striatum uptakes were extracted from SPECT images using MRI- and SPECT-based (SPECT-V and SPECT-T) segmentations respectively. A 2D DenseNet was used to predict HYS of PD, and simultaneously generate deep features (DF). The random forest algorithm was applied to develop models based on DF, RaF, IF and combined features to predict HYS (stage 0, 1 and 2) at year 0 and (stage 0, 1 and ≥ 2) at year 4, respectively. Model predictive accuracy and receiver operating characteristic (ROC) analysis were assessed for various prediction models.

**Results:**

For the diagnostic accuracy at year 0, DL (0.696) outperformed most models, except DF + IF in SPECT-V (0.704), significantly superior based on paired t-test. For year 4, accuracy of DF + RaF model in MRI-based method is the highest (0.835), significantly better than DF + IF, IF + RaF, RaF and IF models. And DL (0.820) surpassed models in both SPECT-based methods. The area under the ROC curve (AUC) highlighted DF + RaF model (0.854) in MRI-based method at year 0 and DF + RaF model (0.869) in SPECT-T method at year 4, outperforming DL models, respectively. And then, there was no significant differences between SPECT-based and MRI-based segmentation methods except for the imaging feature models.

**Conclusion:**

The combination of radiomic and deep features enhances the prediction accuracy of PD HYS compared to only radiomics or DL. This suggests the potential for further advancements in predictive model performance for PD HYS at year 0 and year 4 after first diagnosis using ^123^I-Ioflupane SPECT images at year 0, thereby facilitating early diagnosis and treatment for PD patients. No significant difference was observed in radiomics results obtained between MRI- and SPECT-based striatum segmentations for radiomic and deep features.

**Supplementary Information:**

The online version contains supplementary material available at 10.1186/s40658-024-00651-1.

## Introduction

The prevalence of Parkinson’s disease (PD) is rapidly increasing worldwide, becoming the second most common neurodegenerative disease [1] and imposing a large economic and societal burden [2]. PD is a progressive neurodegenerative disease characterized primarily by the death of dopaminergic neurons in the nigrostriatal pathway, leading to a substantial reduction of the presynaptic dopamine transporter [3]. The use of medication, such as levodopa [4], dopamine agonists [5], and Monoamine oxidase B inhibitors [6], can effectively alleviate the PD symptoms. Deep brain stimulation surgery is another viable approach to improve motor function and symptom control in certain cases [7, 8]. However, although these treatments are efficient on relieving PD symptoms and improving the life quality of patients, there is no treatment for definitive cure so far. Thus, an accurate and timely diagnosis and classification of PD are essential for an early intervention to slow down the disease progression, which can indeed be a challenging process for clinicians.

Clinicians typically identify PD based on the neurologic examination and motor symptoms, such as tremor, rigidity, bradykinesia, gait and balance problems. On the other hand, single photon emission computed tomography (SPECT) can be used for early diagnosis of PD, even before symptoms occur. For example, SPECT radiotracers targeting to the dopamine transporter, i.e., ^123^I-ioflupane (^123^I-FP-CIT, DAT-SPECT) and ^99m^Tc-TRODAT-1, have been used to detect the deficiency of striatal dopamine with high sensitivity [9]. Additionally, SPECT also assists to discriminate PD from other Parkinsonism diseases, as well as improve progression tracking [10, 11]. ^123^I-ioflupane SPECT is now more commonly used in developed countries for PD. Its uptake is significantly reduced in striatum in PD, which could be a quantitative biomarker of neuronal degeneration. It also shows high correlation with disease severity, such as Hoehn and Yahr Stage (HYS) [12, 13], which is a widely accepted measure of the severity and disability level of PD.

In recent years, there has been a significant increase in the use of radiomics for disease diagnosis even in early stages. Radiomics provides a deeper understanding of disease biology by extracting a large number of features from images [14, 15]. More recently, the heterogeneity and texture analysis have also been applied to DAT-SPECT, with improved quality of PD recognition and clinical decisions [16, 17].

On the other hand, deep learning (DL) is promising for medical imaging and have been applied in various medical applications during the last few years. Various network architectures have been proposed and utilized to enhance the precision of PD prediction based on multi-modality imaging data [18–21]. Moreover, deep features combined with machine learning classifiers have been harnessed to bolster the efficacy of prediction models in tumors [22, 23], but yet to be applied in PD.

Therefore, in this study, we aimed to develop prediction models with features extraction from radiomics and DL using ^123^I-Ioflupane SPECT images from the Parkinson’s Progressive Marker Initiative (PPMI) database [24, 25], a multicenter international study platform for PD. We used radiomic features (RaF), deep features (DF), and conventional imaging features (IF) extracted from baseline SPECT images by three segmentation methods to predict HYS at year 0 and year 4 after the first diagnosis of PD. Meanwhile, a DL model was also built for the same prediction task.

## Methods

### Patients dataset

**Fig. 1:**
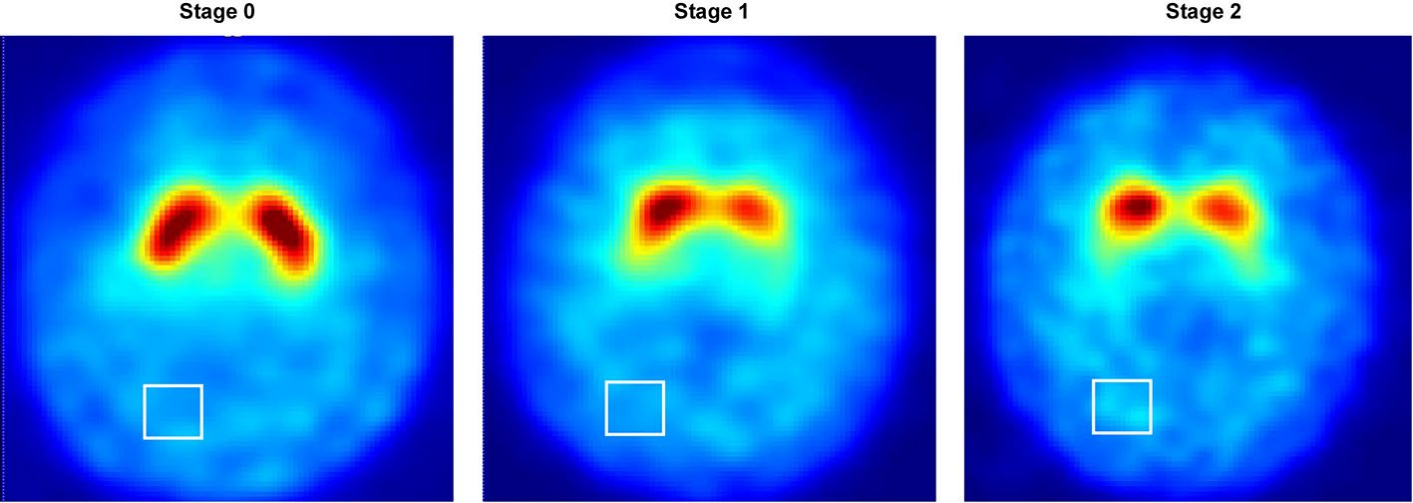
^123^I-Ioflupane SPECT images at 0–2 HYS stages of PD at year 0 after first diagnosis. The white box corresponds to the reference region in the occipital cortex with non-specific uptake and excluding ventricular regions

We analyzed DAT images from PPMI database (www.ppmiinfo.org/data [22, 23]), a multicenter international study platform for PD. Given our focus on patients in the early to moderate stages of PD, individuals aged 30 years or older, with baseline (year 0) Hoehn and Yahr (H&Y) scale of 0, 1, or 2 as well as available HYS information at year 4, and having DAT-SPECT and T1-weighted MRI images at year 0, were included in the study. All PD patients underwent standard treatment, including levodopa and dopamine agonists. With the selection criteria, 161 subjects were enrolled, including 123 PD patients (57 early and 66 moderate cases) and 38 normal controls at year 0, while 121 PD patients (20 early and 101 moderate-advanced cases) and 40 normal controls at year 4. All subjects selected had undergone DAT-SPECT and a high-resolution 3 T MRI scan at baseline (year 0). The required data also included HYS information at both baseline and year 4. Table 1 shows the patients characteristics at year 0 and year 4 after first diagnosis respectively. Figure 1 shows baseline DAT-SPECT images corresponding to different HYS stages of PD. Notably, two patients who were initially assessed as being at HYS stage 2 and stage 1 at year 0 were re-evaluated at year 4 and found to be downgraded to stage 0. Four patients initially classified as stage 1 at year 0 showed disease progression and were re-evaluated as stage 3 at year 4. Additionally, one patient at stage 2 at year 0 progressed to stage 5 at year 4. Thirty-four patients at stage 1 at year 0 advanced to stage 2 at year 4, while the others stay at the same stages (Supplementary Table S1). In this study, a 3-class prediction model was established based on 3 disease stage: HYS (stage 0, 1 and 2) at year 0 and (stage 0, 1 and ≥ 2) at year 4. Stage 0 here indicated normal cases, stage 1 was for mild PD, and stage ≥ 2 was for moderate-late PD.

**Table 1:** The patient characteristics in this study

	Year 0	Year 4
Age (Median [Min, Max], yr)	61 [31, 82]	65 [35, 86]
Gender (Male /Female)	107/54	107/54
Diagnosis (HC/PD)	38/123	40/121
HYS stages	Stage 0 (HC) = 38 Stage 1 = 57 Stage 2 = 66	Stage 0 (HC) = 40 Stage 1 = 20 Stage ≥ 2 = 101

HC: healthy control; PD, Parkinson’s disease

### Imaging acquisition

All subjects were scanned using a 3 T MRI scanner (TrioTim, Siemens Medical Solutions, Erlangen, Germany) with a body coil. T1-weighted images were acquired with following parameters: 240 × 256 × 176 matrix size, 1 × 1 × 1 mm^3^voxel size; TR = 2300 ms, TE = 3 ms, flip angle = 9°. Attenuation-corrected ^123^I-FP-CIT SPECT reconstructed imaging data were retrieved from the PPMI website. SPECT images were captured on a clinical SPECT/CT scanner (Siemens Medical Solutions, Erlangen, Germany), 3–4 h after the injection of ^123^I-FP-CIT (111–185 MBq). More detailed information about the imaging protocol is available at PPMI website (www.ppmi-info.org/study-design).

### Volume of interest (VOI) segmentation

Three different striatum segmentation methods were evaluated. Firstly, MRI-based method was employed to segment four striatal compartments. We utilized ITK-SNAP [26] to semi-automatically register SPECT to MRI images using rigid transformation and followed by manual adjustment. The SPECT images were further resampled into the same size of MRI images. Four individual striatal compartments, i.e., left caudate (LC), right caudate (RC), left putamen (LP), and right putamen (RP), were manually segmented from T1-weighted MRI images by a nuclear medicine radiologist with ten years of experience. Then, the segmented maps were applied to the co-registered SPECT images and VOIs of four striatal compartments from DAT-SPECT were obtained.

Two other segmentation methods were based on SPECT images. One was the standard thresholding method (SPECT-T), using 67% of the maximum intensity in striatum region for striatum segmentation [27, 28]. Another one was the fixed striatum volume (SPECT-V) approach, where a seed-growing method using ITK-SNAP software (Version 3.8.0, http://openiconlibrary.sourceforge.net/) was employed to acquire voxels with the highest intensity in the bilateral striatum from the SPECT image, with the volume of the striatum based on the MRI-derived VOI. Thus, the volumes of VOI are the same for SPECT-V and MRI-based segmentation methods, but their shapes may not be necessarily the same.

### Radiomics and conventional imaging feature extraction

After striatum segmentation, a total of 944 RaF were extracted, including 14 shape-based features, 18 first order intensity features, 75 texture features, and 837 transformed features using the 3D slicer software (Version 5.1.0, https://www.slicer.org/). The texture features encompassed first-, second- and higher-order textural characteristics, capturing information related to the intensity and spatial distribution of radiotracer uptake.

IF including striatal binding ratio (SBR, Eq. (1)), asymmetry index (%ASI, Eq. (2)), maximum (SUV_max_) and mean (SUV_mean_) standardized uptake values were acquired. In the MRI-based segmentation method, a total of 25 IF were extracted for four striatal compartments. While for SPECT-based segmentation method, 10 IF were extracted since only 2 striatal compartments, i.e., left and right striatum, can be segmented (Supplementary Table S2).

1 $$SBR=\frac{{{Mean\_Counts}_{VOI}-Mean\_Counts}_{background}}{{Mean\_Counts}_{background}}$$

2 $$ASI=\left|\frac{{SBR}_{left VOI }-{SBR}_{right VOI}}{{SBR}_{left VOI }+{SBR}_{right VOI}}\right|\times 100\%$$

where the VOI indicates the individual striatal compartments and left/right striatum respectively, depending on the segmentation methods. The background area was chosen from a reference region in the occipital cortex with non-specific uptake and excluding ventricular regions (Fig. 1).

### Deep learning and deep features extraction

**Fig. 2:**
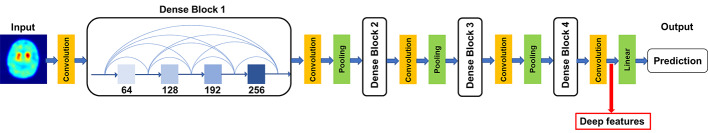
The architecture of DenseNet for HYS prediction and DF extraction

A 2D DenseNet [29] (Fig. 2) was implemented to predict HYS and extract DF for year 0 and year 4, respectively. An additional set of 161 cases from PPMI was employed to train the network (Supplementary Table S3). The five selected axial slices of the 161 cases, i.e., a total of 805 2D slices (109 × 91) with the highest striatal uptake of the SPECT images, and HYS results at year 0 and year 4 after first diagnosis were used as individual inputs to train and validate (8:2) the DenseNet. In the testing phase, the target 161 cases for DF extraction, same as those used in the radiomics study, were input to the trained DenseNet model. Then, the feature maps (264 × 1) before the last output layer of the five slices for each patient were extracted and averaged as DF.

### Feature selection and model construction

**Fig. 3:**
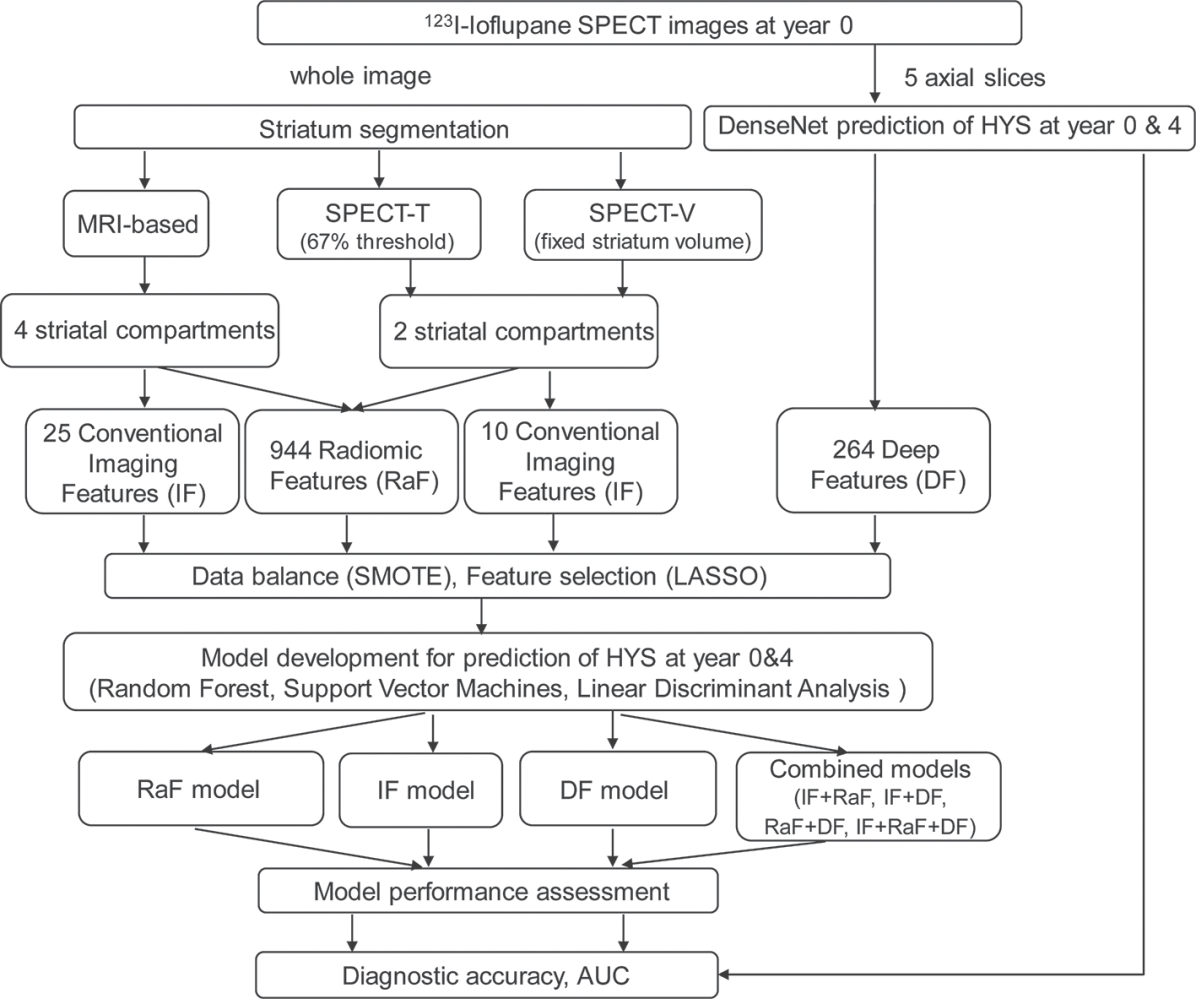
The workflow of this study

Following the extraction of features, the prediction models were constructed. The data was splitting into training data (113 cases) and testing data (48 cases) after data normalization [30], which involved subtracting the mean from each feature vector and then dividing the results by the standard deviation of that feature. The Least Absolute Shrinkage and Selection Operator (LASSO) [31] was employed to select the most associated features with a 10-fold cross validation. To mitigate the imbalance between data from normal and PD patients, the Synthetic Minority Over-sampling Technique was applied, ensuring a more equitable representation of the two classes in the training data. For the feature-based classification task, three different algorithms were evaluated: Random Forest (RF), Support Vector Machines (SVM), and Linear Discriminant Analysis (LDA). Each algorithm was assessed using a 5-fold cross-validation, ensuring the models’ robustness and generalizability. The modeling progress was repeated 10 times to reduce the sampling errors. There were 7 prediction models based on different combination of features, i.e., IF model, RaF model, DF model, IF + RaF model, IF + DF model, RaF + DF model, and IF + RaF + DF model to predict HYS at year 0 and year 4, respectively. Model performance was evaluated in testing set using the Area Under the receiver operating Curve (AUC) and accuracy (95% confidence level). A summary flow chart of the whole study is shown in Fig. 3.

### Statistical analysis

All statistical analyses and modeling were conducted in R software (version 4.2.1, R Foundation for Statistical Computing, Vienna, Austria). A 2-tailed paired t-test was employed to compare the performance between groups with Bonferroni correction for multiple comparison. A p-value < 0.05 was considered significant.

## Results

### Prediction of HYS at Year 0

The details of features selected from LASSO for different models at year 0 and year 4 are showed in Supplementary Figures S1-S2. Averaging the accuracy and AUC for year 0 and year 4, RF was identified as the most effective method among the 3 machine learning algorithms for model development (RF vs. LDA vs. SVM: 0.77 vs. 0.71 vs. 0.73 for accuracy, RF vs. LDA vs. SVM: 0.83 vs. 0.0.83 vs. 0.79 for AUC; Supplementary Figure S3), and thus was used for further classifying different features.

**Fig. 4:**
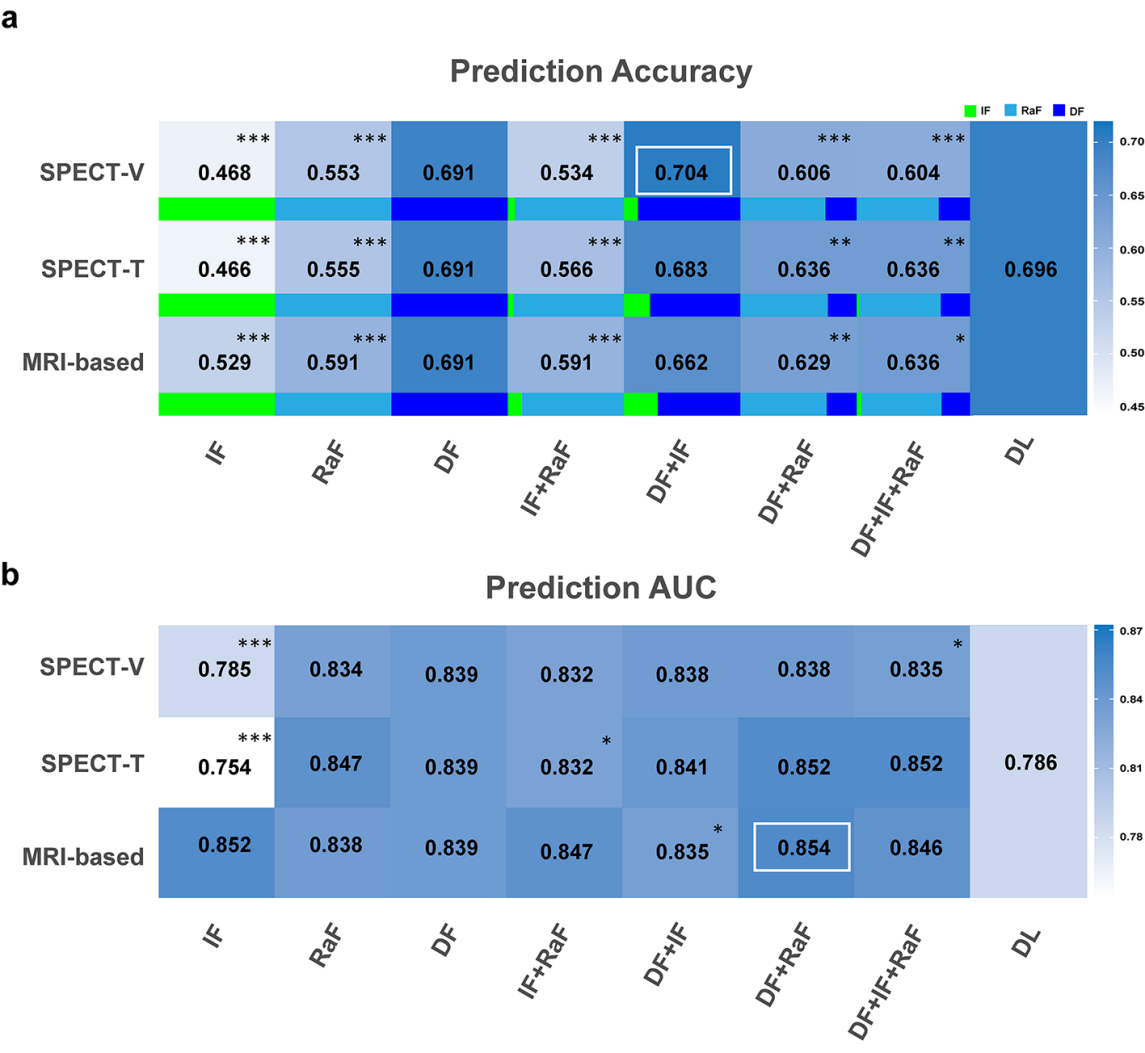
The (**a**) accuracy and (**b**) AUC values of prediction models of HYS at year 0 after first diagnosis. The white box corresponds to the highest value, and the differences between this highest value and those of other models were analyzed (not applicable to the DL model). The proportion of IF (green), RaF (light blue) and DF (dark blue) in each model was shown. ^*^P < 0.05, ^**^P < 0.01, ^***^P < 0.001

Figure 4a shows the mean accuracy of different prediction models of HYS at year 0. The accuracy of DL (0.696) is higher than other models except for DF + IF model in SPECT-V (0.704). The accuracy of DF + IF model in SPECT-V is significantly higher than most of the other models. No significant differences were observed between SPECT-based and MRI-based segmentation methods except for the IF model and IF + RaF model in SPECT-V (Table 2), where MRI-based segmentation methods show better accuracy as compared to SPECT-based segmentations.

Figure 4b shows the mean AUC of all classes for prediction models of HYS at year 0. The mean AUC of DF + RaF model (0.854, sensitivity 66.6%, specificity 80.0%) in MRI-based method is the highest, and it is significantly higher than those of IF models. There are also no significant differences between SPECT-based and MRI-based segmentation methods except for the IF models (Table 2). Additionally, the AUC of DL model (0.786) is less than those of other models except for IF models.

**Table 2:** P values for prediction accuracy and AUC at year 0 after first diagnosis for different segmentation methods

Models	Accuracy			AUC		
	MRI-based vs. SPECT-T	MRI-based vs. SPECT-V	SPECT-T vs. SPECT-V	MRI-based vs. SPECT-T	MRI-based vs. SPECT-V	SPECT-T vs. SPECT-V
DF + RaF	1	0.921	0.588	1	0.106	0.169
IF	0.015^**^	0.021^**^	1	0^***^	0^***^	0.054
IF + DF	1	0.344	0.445	1	1	1
IF + RaF	0.685	0.047^**^	0.258	0.469	0.474	1
RaF	0.058	0.153	1	1	1	0.948
RaF + IF + DF	1	0.604	0.532	1	1	0.132

^*^P < 0.05, ^**^P < 0.01, ^***^P < 0.001

### Prediction of HYS at Year 4

**Fig. 5:**
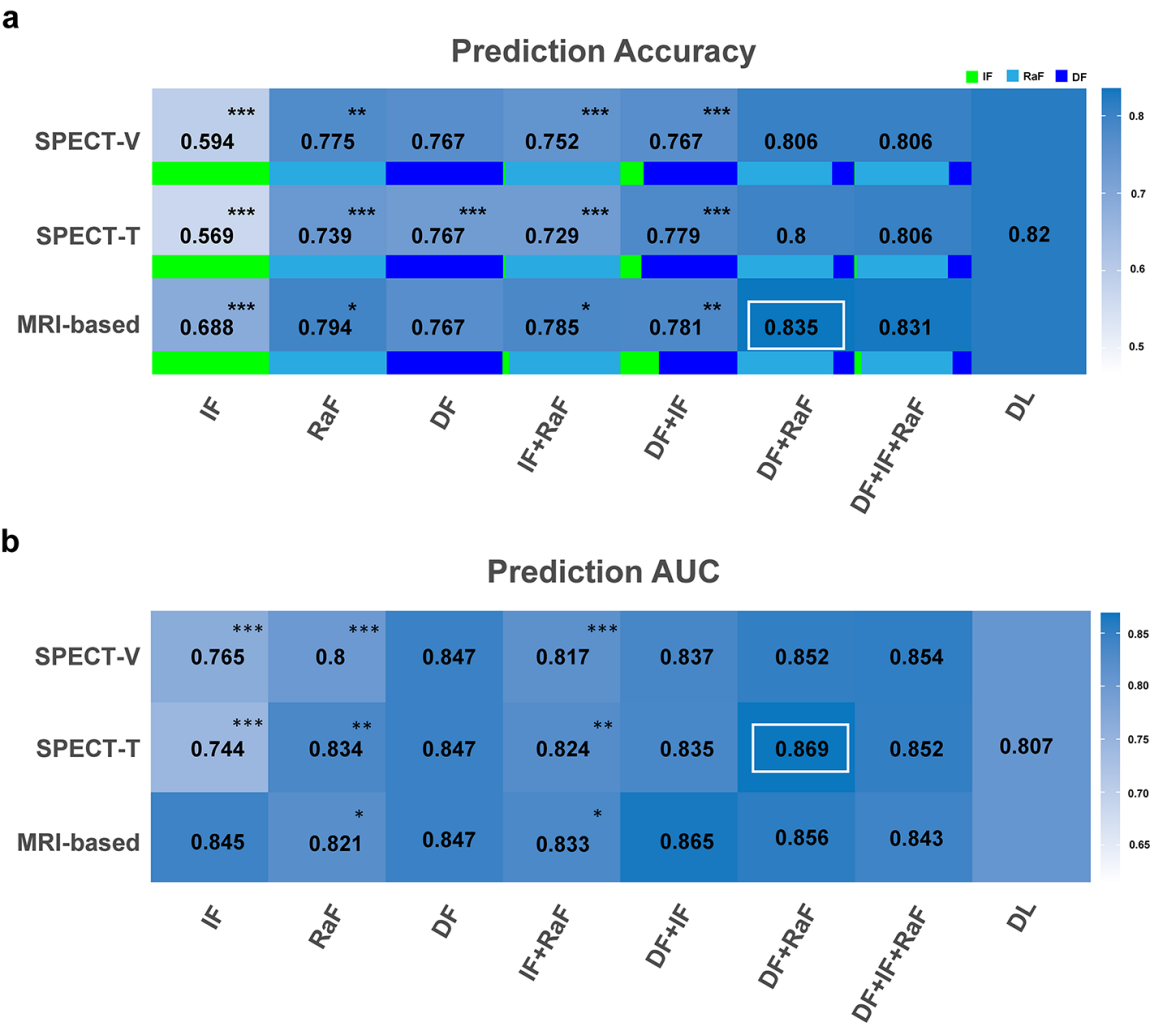
The (**a**) accuracy and (**b**) AUC values of prediction models of HYS at year 4 after first diagnosis. The white box corresponds to the highest value, and the differences between this highest value and those of other models were analyzed. The proportion of IF (green), RaF (light blue) and DF (dark blue) in each model was shown. ^*^P < 0.05, ^**^P < 0.01, ^***^P < 0.001

Figure 5 illustrates the mean accuracy and AUC values of all classes for different prediction models of HYS at year 4. The accuracy of DF + RaF model in MRI-based method is the highest (accuracy 0.835, AUC 0.856, sensitivity 69.6%, specificity 87.8%), significantly better than those of DF + IF, IF + RaF, RaF and IF models (Fig. 5a). The accuracy of DL (0.820) is higher than those models from SPECT-based segmentations. The AUC of DF + RaF model (0.869) in SPECT-T method is the highest, significantly better than those of IF, RaF and IF + RaF models (Fig. 5b). The AUC of DL model (0.807) is lower than that of other models, with the exceptions of the RaF model from SPECT-V segmentation and the IF models from SPECT-based segmentations.

No significant differences were observed between SPECT- and MRI-based segmentation methods except for IF and RaF models for prediction accuracy (Table 3). For AUC, the only significant difference observed between SPECT-based and MRI-based methods was the IF models (Table 3). The mean sensitivity and specificity of prediction models of HYS at year 0 and year 4 after first diagnosis using RF, SVM and LDA classifiers were presented in Supplementary Tables S4 and S5.

**Table 3:** P values for prediction accuracy and AUC at year 4 after first diagnosis for different segmentation methods

Models	Accuracy			AUC		
	MRI-based vs. SPECT-T	MRI-based vs. SPECT-V	SPECT-T vs. SPECT-V	MRI-based vs. SPECT-T	MRI-based vs. SPECT-V	SPECT-T vs. SPECT-V
DF + RaF	0.114	0.532	1	1	1	0.614
IF	0^***^	0.014^**^	0.562	0.002^**^	0.02^**^	0.787
IF + DF	1	0.678	0.421	0.12	0.14	1
IF + RaF	0.128	0.526	0.556	1	0.218	0.673
RaF	0.1	1	0.049^*^	1	0.518	0.153
RaF + IF + DF	0.617	0.327	1	1	0.916	1

^*^P < 0.05, ^**^P < 0.01, ^***^P < 0.001

## Discussion

Radiomics and DL have emerged as promising approaches in the diagnosis and predicting the prognosis of PD. In the current study, we used different segmentation methods, i.e., MRI- and SPECT-based, to predict PD stages at year 0 and year 4 after first diagnosis using baseline (year 0) DAT-SPECT images. Then, DF, IF and RaF were extracted from SPECT images to develop prediction models using RF algorithm. DF has been previously utilized to predict tumor prognosis, showing promising results in cancer research [22, 23, 32]. However, its application in PD has been relatively limited, and there is a scarcity of studies exploring its potential for PD prognosis. The DF + RaF model emerged as the most effective model for predicting PD stages at both year 0 and year 4, with the exception of its accuracy at year 0. This finding may be attributed to the fact that DF + RaF model provide more comprehensive and detailed lesion characteristics compared to IF. DF potentially possess higher-level features from DL algorithms. RaF derived from the analysis of image texture, shape, and intensity, provides a wealth of quantitative information. These features encompass a wide range of lesion characteristics and heterogeneity, enabling a deeper understanding of the underlying biological processes and disease progression. Similar to our study, other researchers have reported that the addition of RaF enhanced predictive performance in PD [16, 33–37].

In general, the mean accuracy of the prediction model at year 4 can be up to 85.4% using DF + RaF model in MRI-based segmentation, higher than those obtained by Hsu et al. (83.2%) [38], Salmanpour et al. (76.1-79.2%) [33] and Huang et al. (26.2-56.4%) [39]. Our dataset predominantly comprises patients in the early to moderate stages, while other studies include a higher proportion of late-stage patients, classifying normal, mild and severe (6:49:147 patients) [38], or mild, intermediate and severe (51:43:39 patients) [33], or stage I-V (22:27:53:87:7 patients) [39]. The variations in patient distribution could potentially impact the comparison of model accuracies. In future investigations, we aim to integrate a substantial amount of late-stage patient data to be compared with existing studies. Although the accuracy and AUC of DF + RaF model at year 4 exceed 0.8 with specificity > 0.8, the sensitivity is generally < 0.7. While the model effectively identifies true negative cases, it may misclassify some high-stage patients as lower-stage, e.g., inability to detect subtle striatal uptake defects in some PD patients. This limitation may be attributed to several factors. The relatively small and imbalanced training dataset (Table 1) limits the models’ performance. Additionally, the selected features may not be able to fully discriminate different classes. In the future, expanding the sample size [40], feature engineering [41] and more machine learning algorithms [42] could be used to enhance the prediction performance.

In our study, we observed that the accuracy of the DL method alone was 82%, which was slightly lower compared to the accuracy achieved by the DF + RaF model based on MRI segmentation. The AUC of DL model is also lower than other models except for RaF models in SPECT-V and IF models. This could be attributed to the utilization of the currently used 2D DenseNet network, which is constrained in capturing spatial information across axial slices as compared to a 3D network. Nevertheless, DL retains several advantages in medical imaging analysis, including automated feature learning, no manual segmentation needed, and end-to-end learning [43, 44]. Additionally, the accuracy obtained using the DL method in our study was also lower than the results reported by Huang et al. (85.5%) [18]. This discrepancy could be attributed to several factors, such as differences in the datasets, imaging protocols, model architectures, and training methodologies used in the two studies.

This project concentrates on predicting HYS classification from SPECT images without considering additional factors, such as patients’ age, gender, education level, and treatment plans. The effects of these factors could be investigated by stratifying the studied population, but it is beyond the scope of this study due to the limited sample size. Our predictive results represent the disease classification in the fourth year following conventional medication treatment for PD patients. This aids in identifying patients sensitive to current treatment regimens. For those predicted to have poor treatment outcomes and disease progression, new medications or alternative treatment methods could be considered, which is valuable for clinical treatment decision-making. Furthermore, predicting HYS at year 0 based on SPECT images not only provides crucial insights into the initial stage of PD but also enhances early diagnosis. Additionally, it enables a more profound understanding of the disease’s severity at its onset [18, 36, 38]. Consequently, it contributes to a more effective disease management and improved patient outcomes, addressing the pressing need for personalized and timely therapeutic strategies in PD care.

Utilizing images from year 0 to predict HYS classification at both year 0 and year 4 holds significant importance. This predictive approach is crucial because it allows for the anticipation of the disease’s severity at the outset (year 0) and its progression over a 4-year period. This information is invaluable for clinicians as it not only aids in early diagnosis but also provides a foundation for devising tailored treatment plans and interventions, ultimately contributing to a more effective disease management and improved patient outcomes. Notably, our results show that the prediction efficacy of HYS at Year 4 is superior to that at Year 0 using ^123^I-Ioflupane SPECT images at year 0, with higher accuracy and AUC values. The highest mean accuracy of the prediction model at year 0 in our study was < 80%, and most of the models had accuracies under 70%, including the DL method. These results indicate that accurately predicting PD stages at year 0 based on the available data was challenging, and the models’ performances were generally modest. We hypothesized that DAT reduction reflected from SPECT images occurs earlier than neurologic changes [3, 45], yet more data are warranted to validate our conclusions. Furthermore, factors such as early-stage disease assessment, potential patient population heterogeneity, and the restricted amount of information accessible at the baseline could collectively contribute to the comparatively lower accuracy observed in the results.

Additionally, no significant differences were observed between MRI-based and SPECT-based segmentation methods for radiomics models to predict HYS in this study. Meanwhile, SPECT-V also requires the information from MRI images, including the segmentation of the four parts of the striatum based on the MRI data. Thus, we recommend the SPECT-based segmentation method for further radiomics studies in PD due to its ease of operation, particularly with SPECT-T. Another research group [36] reported that the RaF model performance based on SPECT images segmented using MRI was superior to the model employing SPECT-based segmentation. One potential reason is the variation in SPECT-based segmentation methods employed in the two studies. We used 3D SPECT images directly to segment, while they converted 3D SPECT image to 2D images, and then constructed 3D VOI through 2D images. Moreover, it is essential to consider that the predicted task and assessment may be different between the two studies. The current study employed HYS as the metric, whereas Unified Parkinson Disease Rating Scale was used in their study. Deep learning-based striatum segmentation based on SPECT images is also feasible [46] (https://ieeexplore.ieee.org/document/10525203) to reduce the inter/intra operator variations and clinical burden for radiomics analysis.

Despite of the promising outcome, this study also has several limitations. As a proof-of-concept methodology study, the patient population of the study cohort was relatively small, with only 161 cases involved. It could limit the generalizability of the findings to a larger population. Moreover, the distribution of different PD stages in the population was imbalanced, particularly for year 4 (Stage 0: Stage 1: Stage ≥ 2 = 40: 20: 101). Therefore, to ensure the reproducibility and generalizability of our approach, it is essential to validate the findings using a larger and more diverse dataset. Additionally, we only extracted image-based features, e.g., DF, RaF and IF, to develop the prediction models. We did not investigate other features, such as age at PD diagnosis, symptoms, medication history, family history, etc., during the model developments. Finally, our study utilized a 2D DenseNet network for predicting PD stages, and the use 3D DL models might further improve the prediction accuracy.

## Conclusion

Radiomics based on baseline DAT-SPECT images is promising for predicting PD HYS stages at both year 0 and year 4 after first diagnosis. The use of deep features has the potential to further improve the radiomics performance. No significant difference was observed between MRI- and SPECT-based striatum segmentations for radiomics-based models. Additionally, the baseline DAT-SPECT image exhibits superior predictive capability for PD HYS at year 4 compared to that at year 0.

### Electronic supplementary material

Below is the link to the electronic supplementary material.

Supplementary Material 1

## Data Availability

All results are provided in the manuscript and its supplementary information files. PPMI data can be freely downloaded from www.ppmi-info.org/data.

## Abbreviations

PD: Parkinson’s disease
SPECT: Single photon emission computed tomography
^123^I-FP-CIT: ^123^I-ioflupane
HYS: Hoehn-Yahr stages
DL: Deep learning
RaF: Radiomic features
DF: Deep features
IF: Conventional imaging features
VOI: Volume of interest
LC: Left caudate
RC: Right caudate
LP: Left putamen
RP: Right putamen
SBR: Striatal binding ratio
%ASI: Asymmetry index
SUVmax: Maximum standardized uptake values
SUVmean: Mean standardized uptake values
LASSO: Least Absolute Shrinkage and Selection Operator
RF: Random Forest
SVM: Support Vector Machines
LDA: Linear Discriminant Analysis
ROC: Receiver Operating Characteristic
AUC: Area Under the receiver operating Curve
