# Supplementary material for: Radiomics incorporating deep features for predicting Parkinson’s disease in 123I-Ioflupane SPECT
Source: EJNMMI Phys. 2024 Jul 10;11:60. doi: 10.1186/s40658-024-00651-1 (PMC11236833; doi:10.1186/s40658-024-00651-1)
Supplement: Supplementary file 1 — Supplementary Material 1 [file 40658_2024_651_MOESM1_ESM.docx]

**Radiomics incorporating deep features for predicting Parkinson’s disease in ^123^I-Ioflupane SPECT**


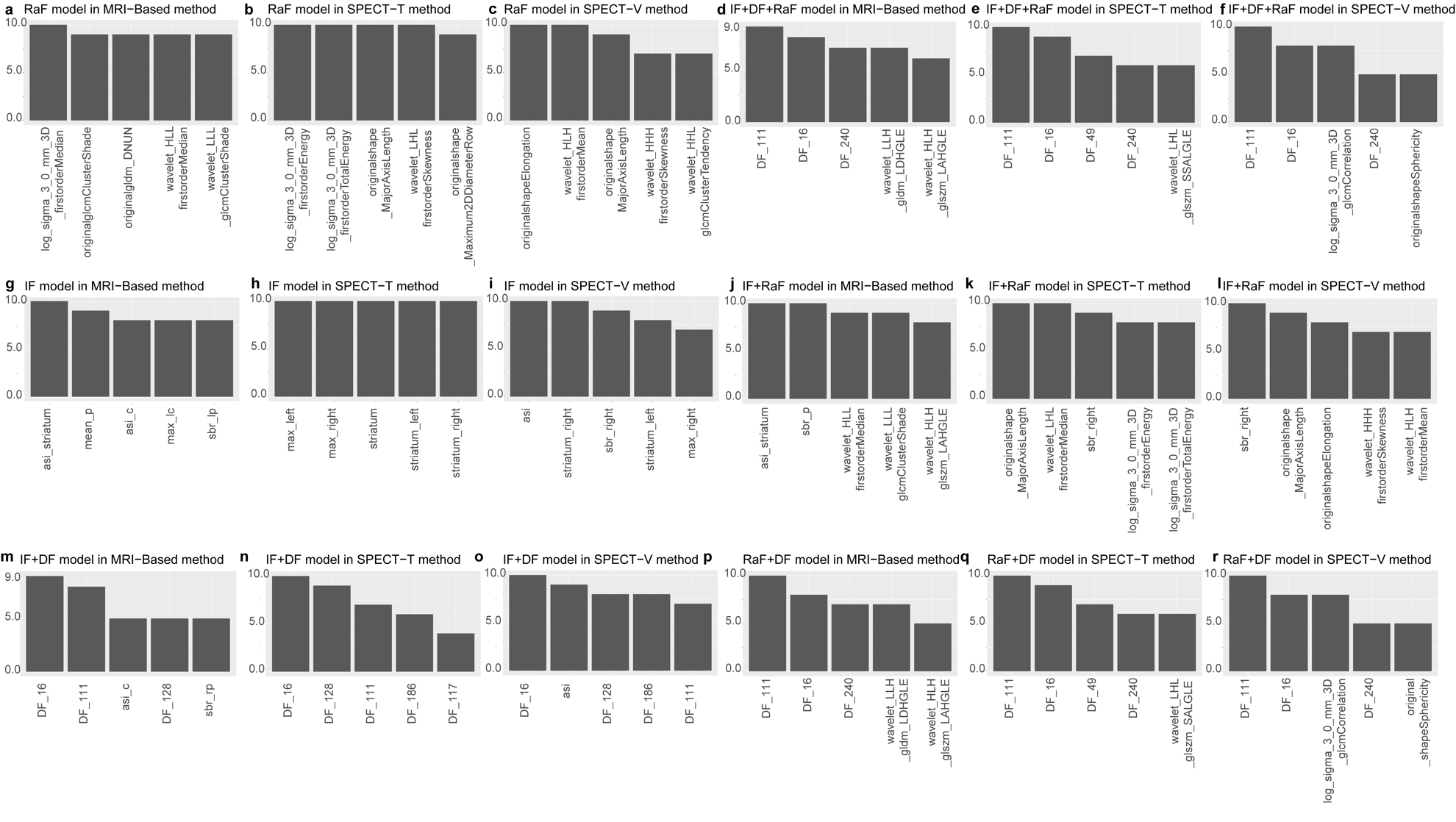


**Figure S1** A multi-panel bar graph showing the frequencies of features (top 5) in each model at year 0 after first diagnosis. (a-c) RaF models; (d-f) IF+DF+RaF models; (g-i) IF models; (j-l) IF+RaF models; (m-o) IF+DF models; (p-r) RaF+DF models in MRI-based, SPECT-T and SPECT-V segmentation methods respectively.

**
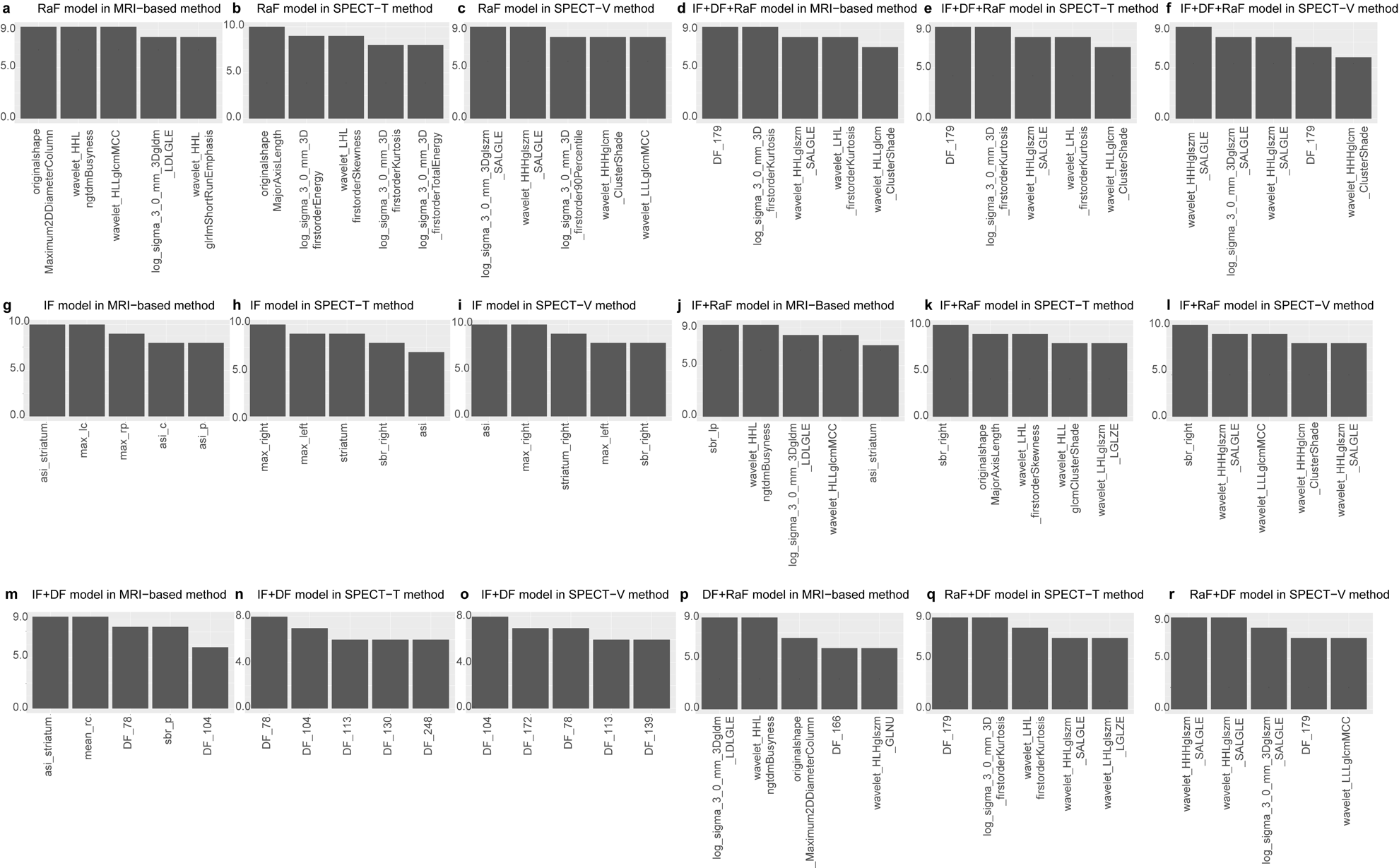
**

**Figure S2** A multi-panel bar graph showing the frequencies of features (top 5) in each model at year 4 after first diagnosis. (a-c) RaF models; (d-f) IF+DF+RaF models; (g-i) IF models; (j-l) IF+RaF models; (m-o) IF+DF models; (p-r) RaF+DF models in MRI-based, SPECT-T and SPECT-V segmentation methods respectively.

**
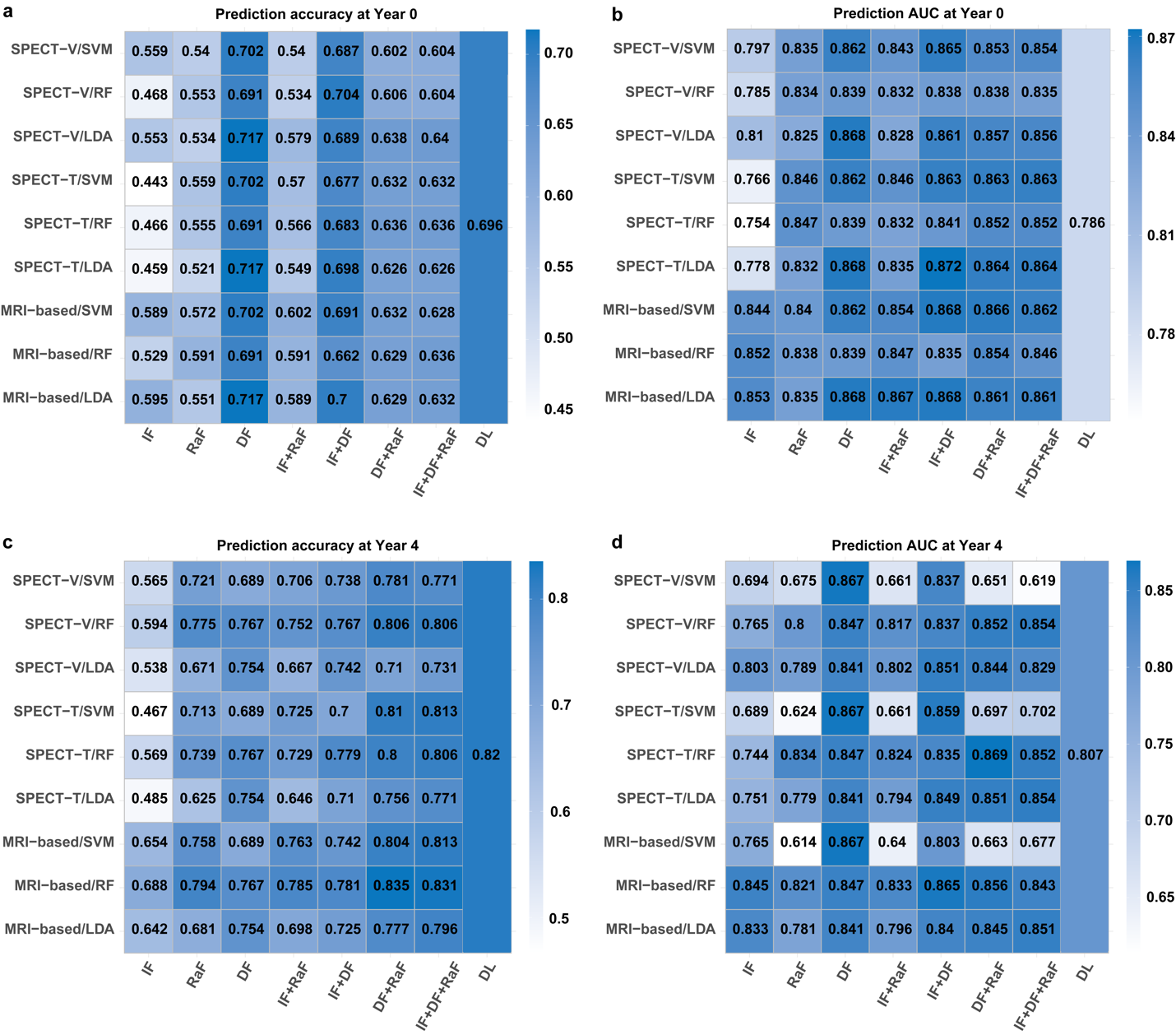
Figure S3** The (a, c) accuracy and (b, d) AUC values of prediction models of HYS at year 0 and year 4 after first diagnosis using RF, SVM and LDA classifiers.

**Table S1** The Hoehn and Yahr Stage (HYS) of the study population at year 0 and year 4.

| **HYS at Year 0** | **HYS at Year 4** | **Patients No.**  **(n = 161)** |
| --- | --- | --- |
| Stage 0 | Stage 0 | 38 |
| Stage 1 | Stage 1 | 18 |
| Stage 1 | Stage 0 | 1 |
| Stage 1 | Stage 2 | 34 |
| Stage 1 | Stage 3 | 4 |
| Stage 2 | Stage 2 | 55 |
| Stage 2 | Stage 0 | 1 |
| Stage 2 | Stage 1 | 2 |
| Stage 2 | Stage 3 | 3 |
| Stage 2 | Stage 4 | 4 |
| Stage 2 | Stage 5 | 1 |

**Table S2** The IF extracted from striatum using MRI- and SPECT-based segmentation.

| **IF feature category** | **MRI-based segmentation**  **(n = 25)** | **SPECT-based segmentation**  **(n = 10)** |
| --- | --- | --- |
| **Mean counts** | right caudate | left striatum |
|  | left caudate | right striatum |
|  | right putamen | striatum |
|  | left putamen |  |
|  | left striatum |  |
|  | right striatum |  |
|  | caudate |  |
|  | putamen |  |
|  | striatum |  |
| **Maximum counts** | right caudate | left striatum |
|  | left caudate | right striatum |
|  | right putamen | striatum |
|  | left putamen |  |
| **SBR** | right caudate | left striatum |
|  | left caudate | right striatum |
|  | right putamen | striatum |
|  | left putamen |  |
|  | left striatum |  |
|  | right striatum |  |
|  | caudate |  |
|  | putamen |  |
|  | striatum |  |
| **ASI** | caudate | striatum |
|  | putamen |  |
|  | striatum |  |

**Table S3** The characteristics of the additional 161 datasets for DL training.

|  | Year 0 | Year 4 |
| --- | --- | --- |
| Age (Median [Min, Max]，yr) | 62 [30, 84] | 66 [34, 88] |
| Gender (Male /Female) | 94/67 | 94/67 |
| Diagnosis (HC/PD) | 47/114 | 48/113 |
| HYS stages | Stage 0 (HC) = 48  Stage 1 = 59  Stage 2 = 54 | Stage 0 (HC) = 48  Stage 1 = 29  Stage ≥2 = 84 |

**Table S4** The sensitivity and specificity values of various prediction models of HYS at year 0.

| **Models** | **Segmentation methods** | **Sensitivity** | | | **Specificity** | | |
| --- | --- | --- | --- | --- | --- | --- | --- |
|  |  | **SVM** | **RF** | **LDA** | **SVM** | **RF** | **LDA** |
| **DF+RaF** | SPECT-V | 0.642 | 0.646 | 0.678 | 0.788 | 0.789 | 0.807 |
|  | SPECT-T | 0.669 | 0.676 | 0.667 | 0.804 | 0.806 | 0.800 |
|  | MRI based | 0.664 | 0.666 | 0.666 | 0.801 | 0.800 | 0.800 |
| **IF** | SPECT-V | 0.582 | 0.494 | 0.584 | 0.773 | 0.725 | 0.768 |
|  | SPECT-T | 0.480 | 0.490 | 0.498 | 0.715 | 0.724 | 0.726 |
|  | MRI based | 0.626 | 0.570 | 0.629 | 0.782 | 0.750 | 0.788 |
| **IF+DF** | SPECT-V | 0.720 | 0.734 | 0.722 | 0.833 | 0.842 | 0.834 |
|  | SPECT-T | 0.706 | 0.715 | 0.729 | 0.824 | 0.830 | 0.838 |
|  | MRI based | 0.717 | 0.698 | 0.726 | 0.833 | 0.818 | 0.838 |
| **IF+RaF** | SPECT-V | 0.578 | 0.579 | 0.618 | 0.755 | 0.755 | 0.780 |
|  | SPECT-T | 0.598 | 0.602 | 0.584 | 0.771 | 0.771 | 0.762 |
|  | MRI based | 0.634 | 0.630 | 0.622 | 0.788 | 0.783 | 0.785 |
| **RaF** | SPECT-V | 0.575 | 0.593 | 0.573 | 0.757 | 0.765 | 0.757 |
|  | SPECT-T | 0.586 | 0.590 | 0.559 | 0.766 | 0.765 | 0.749 |
|  | MRI based | 0.600 | 0.627 | 0.584 | 0.774 | 0.784 | 0.766 |
| **RaF+IF+DF** | SPECT-V | 0.644 | 0.646 | 0.679 | 0.789 | 0.789 | 0.808 |
|  | SPECT-T | 0.669 | 0.676 | 0.667 | 0.804 | 0.806 | 0.800 |
|  | MRI based | 0.661 | 0.673 | 0.668 | 0.799 | 0.804 | 0.802 |
| **DF** | NA | 0.731 | 0.723 | 0.745 | 0.839 | 0.835 | 0.848 |

**Table S5** The sensitivity and specificity values of various prediction models of HYS at year 4.

| **Models** | **Segmentation methods** | **Sensitivity** | | | **Specificity** | | |
| --- | --- | --- | --- | --- | --- | --- | --- |
|  |  | **SVM** | **RF** | **LDA** | **SVM** | **RF** | **LDA** |
| **DF+RaF** | SPECT-V | 0.625 | 0.664 | 0.620 | 0.840 | 0.866 | 0.829 |
|  | SPECT-T | 0.698 | 0.666 | 0.730 | 0.864 | 0.860 | 0.867 |
|  | MRI based | 0.643 | 0.696 | 0.675 | 0.855 | 0.878 | 0.861 |
| **IF** | SPECT-V | 0.487 | 0.487 | 0.487 | 0.760 | 0.764 | 0.761 |
|  | SPECT-T | 0.454 | 0.490 | 0.475 | 0.719 | 0.741 | 0.730 |
|  | MRI based | 0.608 | 0.597 | 0.638 | 0.817 | 0.818 | 0.827 |
| **IF+DF** | SPECT-V | 0.675 | 0.648 | 0.709 | 0.853 | 0.856 | 0.862 |
|  | SPECT-T | 0.677 | 0.664 | 0.679 | 0.841 | 0.863 | 0.849 |
|  | MRI based | 0.681 | 0.654 | 0.684 | 0.856 | 0.858 | 0.857 |
| **IF+RaF** | SPECT-V | 0.542 | 0.598 | 0.596 | 0.782 | 0.816 | 0.806 |
|  | SPECT-T | 0.526 | 0.593 | 0.496 | 0.764 | 0.792 | 0.733 |
|  | MRI based | 0.597 | 0.639 | 0.614 | 0.823 | 0.844 | 0.825 |
| **RaF** | SPECT-V | 0.544 | 0.621 | 0.589 | 0.790 | 0.832 | 0.814 |
|  | SPECT-T | 0.552 | 0.605 | 0.543 | 0.784 | 0.817 | 0.782 |
|  | MRI based | 0.596 | 0.634 | 0.604 | 0.826 | 0.853 | 0.820 |
| **RaF+IF+DF** | SPECT-V | 0.619 | 0.663 | 0.652 | 0.834 | 0.863 | 0.842 |
|  | SPECT-T | 0.704 | 0.680 | 0.726 | 0.866 | 0.865 | 0.871 |
|  | MRI based | 0.641 | 0.672 | 0.686 | 0.856 | 0.871 | 0.868 |
| **DF** | NA | 0.667 | 0.665 | 0.707 | 0.836 | 0.858 | 0.863 |
